# Supplementary material for: Longitudinal analysis within one hospital in sub-Saharan Africa over 20 years reveals repeated replacements of dominant clones of Klebsiella pneumoniae and stresses the importance to include temporal patterns for vaccine design considerations
Source: Genome Med. 2024 May 6;16:67. doi: 10.1186/s13073-024-01342-3 (PMC11073982; doi:10.1186/s13073-024-01342-3)
Supplement: Supplementary file 1 — Additional file 1: Supplementary figures Fig.S1-Fig.S12 and respective figure legends and a description of Tables S1-S6. [file 13073_2024_1342_MOESM1_ESM.pdf]

Supporting materials

Longitudinal analysis within one hospital in sub-Saharan Africa over 20 years reveals repeated replacements of dominant clones of *Klebsiella pneumoniae* and stresses the importance to include temporal patterns for vaccine design considerations

*Eva Heinz[1,2], Oliver Pearse[2,3], Allan Zuza[3], Sithembile Bilima[3], Chisomo Msefula[4], Patrick Musicha[2,3], Patriciah Siyabu[5], Edith Tewesa[5], Fabrice E Graf[2], Rebecca Lester[3,6], Samantha Lissauer[3,7], Jennifer Cornick[3,7], Joseph M Lewis[2,3,7], Kondwani Kawaza[3,4], Nicholas R Thomson[8,9], *Nicholas A Feasey[2,3,10]

1. Department of Vector Biology, Liverpool School of Tropical Medicine, Liverpool, UK
2. Department of Clinical Sciences, Liverpool School of Tropical Medicine, Liverpool, UK
3. Malawi Liverpool Wellcome Programme, Kamuzu University of Health Sciences, Blantyre, Malawi
4. Kamuzu University of Health Sciences, Blantyre, Malawi
5. Queen Elizabeth Central Hospital, Blantyre, Malawi
6. Division of Infection & Immunity, University College London, UK
7. Department of Clinical Infection, Microbiology and Immunology, University of Liverpool, Liverpool, UK
8. Parasites and Microbes Program, Wellcome Sanger Institute, Hinxton, UK
9. London School of Hygiene and Tropical Medicine, London, UK
10. School of Medicine, St Andrews University, UK

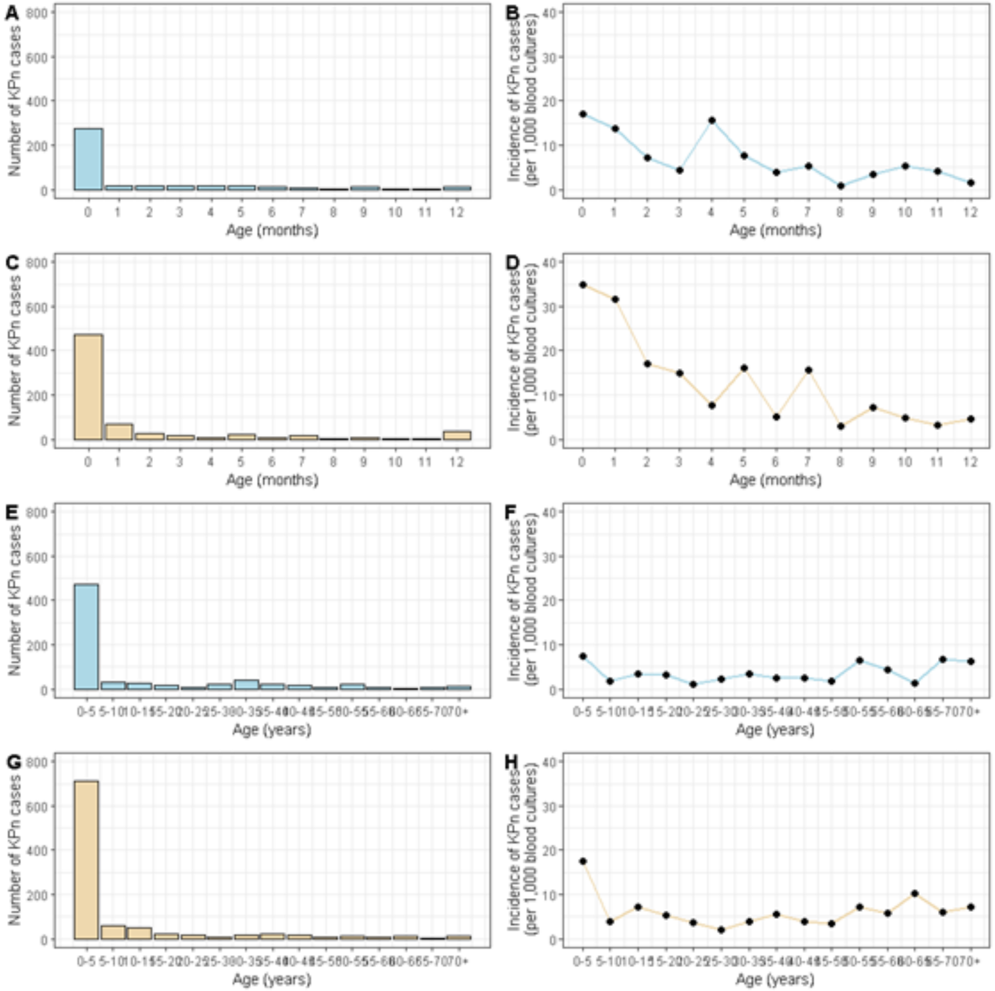
A
Number of KPn cases
Age (months)
B
Incidence of KPn cases (per 1,000 blood cultures)
Age (months)
C
Number of KPn cases
Age (months)
D
Incidence of KPn cases (per 1,000 blood cultures)
Age (months)
E
Number of KPn cases
Age (years)
F
Incidence of KPn cases (per 1,000 blood cultures)
Age (years)
G
Number of KPn cases
Age (years)
H
Incidence of KPn cases (per 1,000 blood cultures)
Age (years)

Fig S1. Age specific *Kpn* crude frequency and blood culture positivity rate. (**A**) Number of *Kpn* cases up to age 12 months (1998-2015). (**B**) Blood culture positivity rate (per 1,000 blood cultures) of *Kpn* cases up to age 12 months (1998-2015). (**C**) Number of *Kpn* cases up to age 12 months (2016-2021). (**D**) Blood culture positivity rate (per 1,000 blood cultures) of *Kpn* cases up to age 12 months (2016-2021). (**E**) Number of *Kpn* cases up to 100 years (1998-2015). (**F**) Blood culture positivity rate (per 1,000 blood cultures) of *Kpn* cases up to 100 years (1998-2015). (**G**) Number of *Kpn* cases up to 100 years (2016-2021). (**H**) Blood culture positivity rate (per 1,000 blood cultures) of *Kpn* cases up to 100 years (2016-2021).

Figure S2

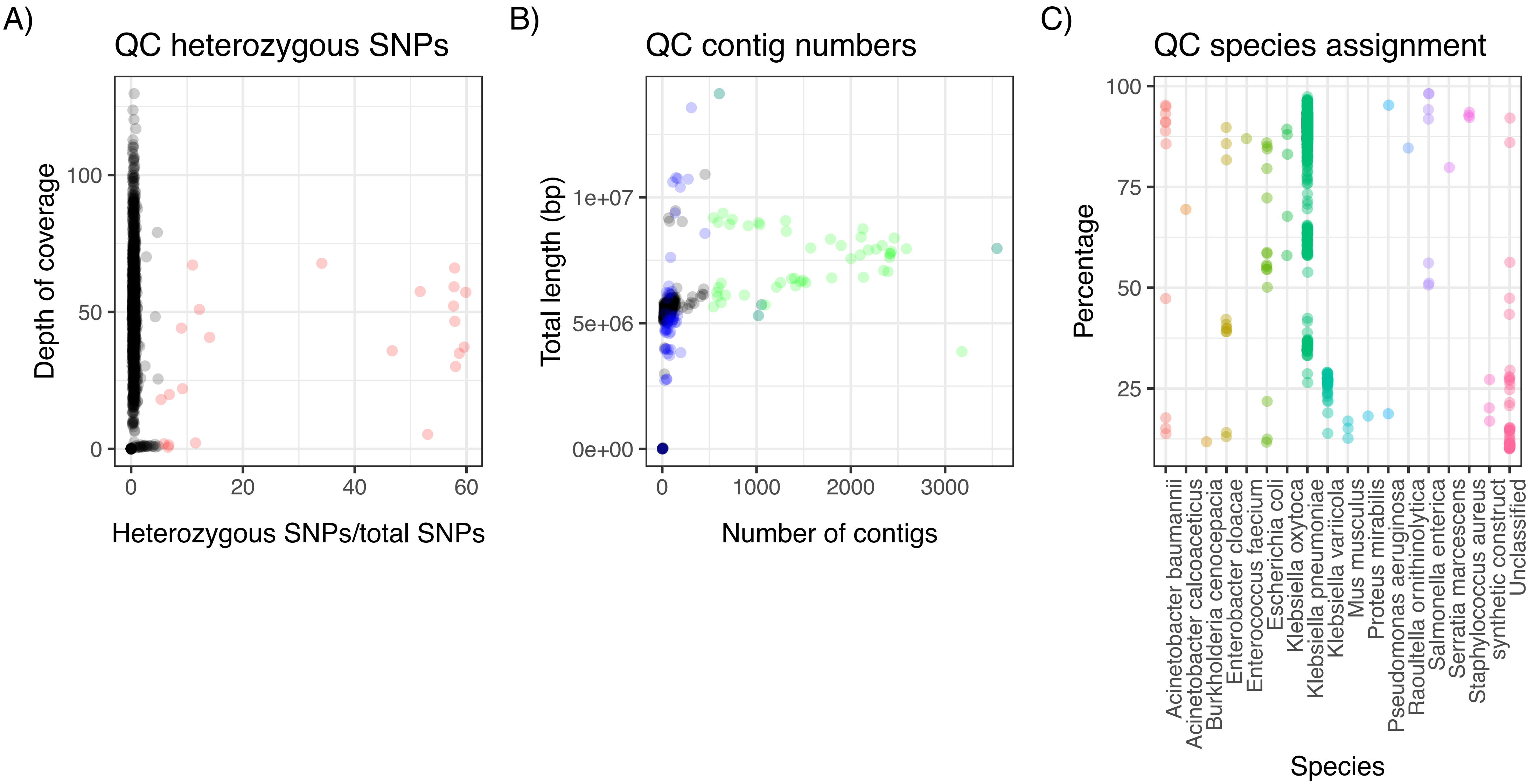
A)
QC heterozygous SNPs
Depth of coverage
100
50
0
0
20
40
60
Heterozygous SNPs/total SNPs
B)
QC contig numbers
Total length (bp)
1e+07
5e+06
0e+00
0
1000
2000
3000
Number of contigs
C)
QC species assignment
Percentage
100
75
50
25
Acinetobacter baumannii
Acinetobacter calcoaceticus
Burkholderia cenocepacia
Enterobacter cloacae
Enterococcus faecium
Escherichia coli
Klebsiella oxytoca
Klebsiella pneumoniae
Klebsiella variicola
Mus musculus
Proteus mirabilis
Pseudomonas aeruginosa
Raoultella ornithinolytica
Salmonella enterica
Serratia marcescens
Staphylococcus aureus
synthetic construct
Unclassified
Species

Fig S2. Quality control of sequencing data. (**A**) The plot shows the distribution of homozygous vs heterozygous SNPs, isolates represented by red points (>5% heterozygous SNPs) were removed as likely consisting of two related strains. (**B**) Number of contigs vs total assembly length is shown; with isolates that passed QC in black, isolates that failed the species composition QC in blue, and isolates with >500 contigs in green, which were also removed. The species assignment of the sequencing reads according to kraken are shown in panel (**C**), where all isolates with more than 10% of a different species were removed.

Fig. S3 Major STs per wards

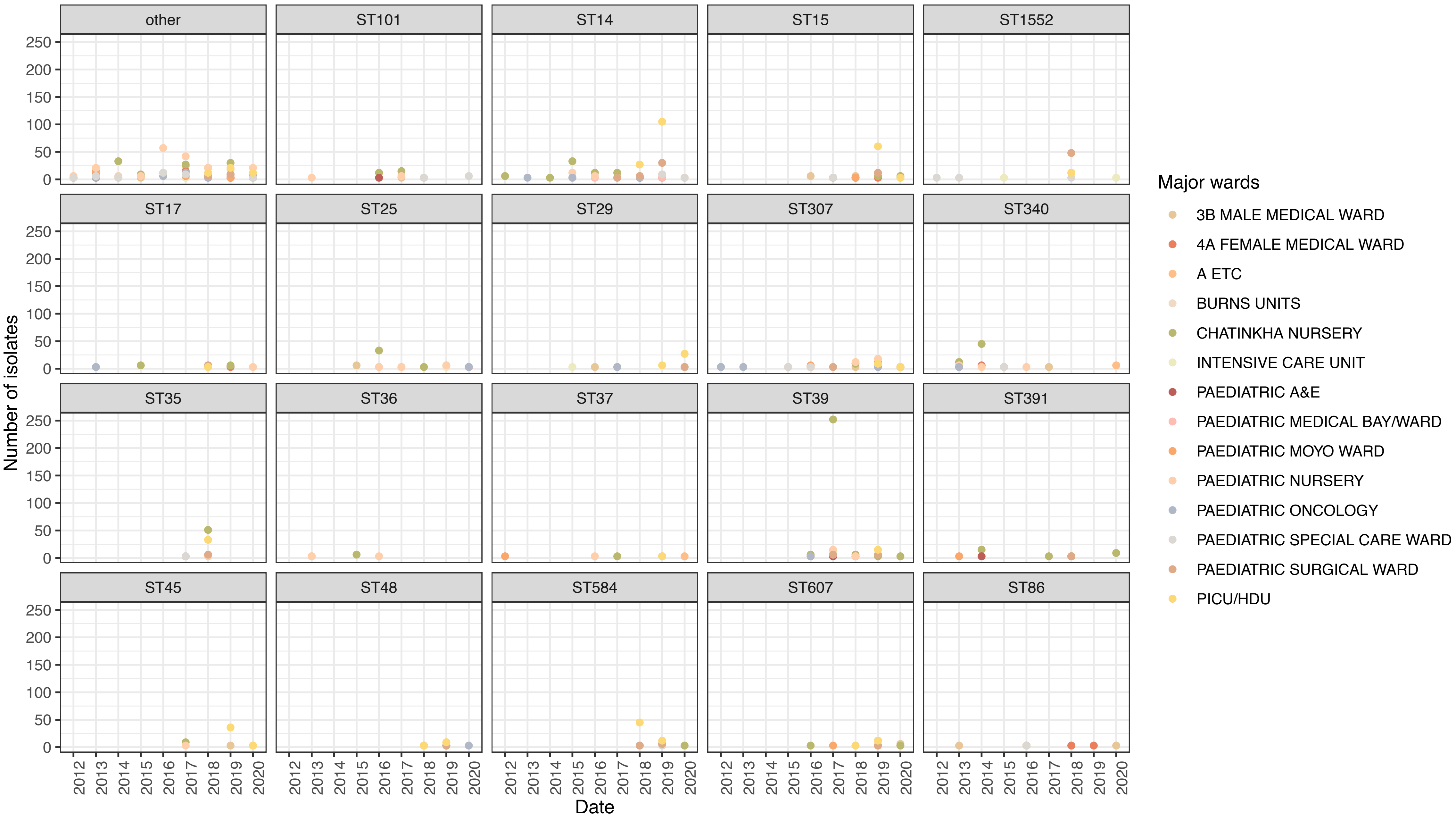

Fig S3. Main ST isolates per wards over time. Each panel shows the number of isolates for the relevant ST as in the legend. The number of isolates per year are stratified by the major wards as in the figure legend.

Figure S4

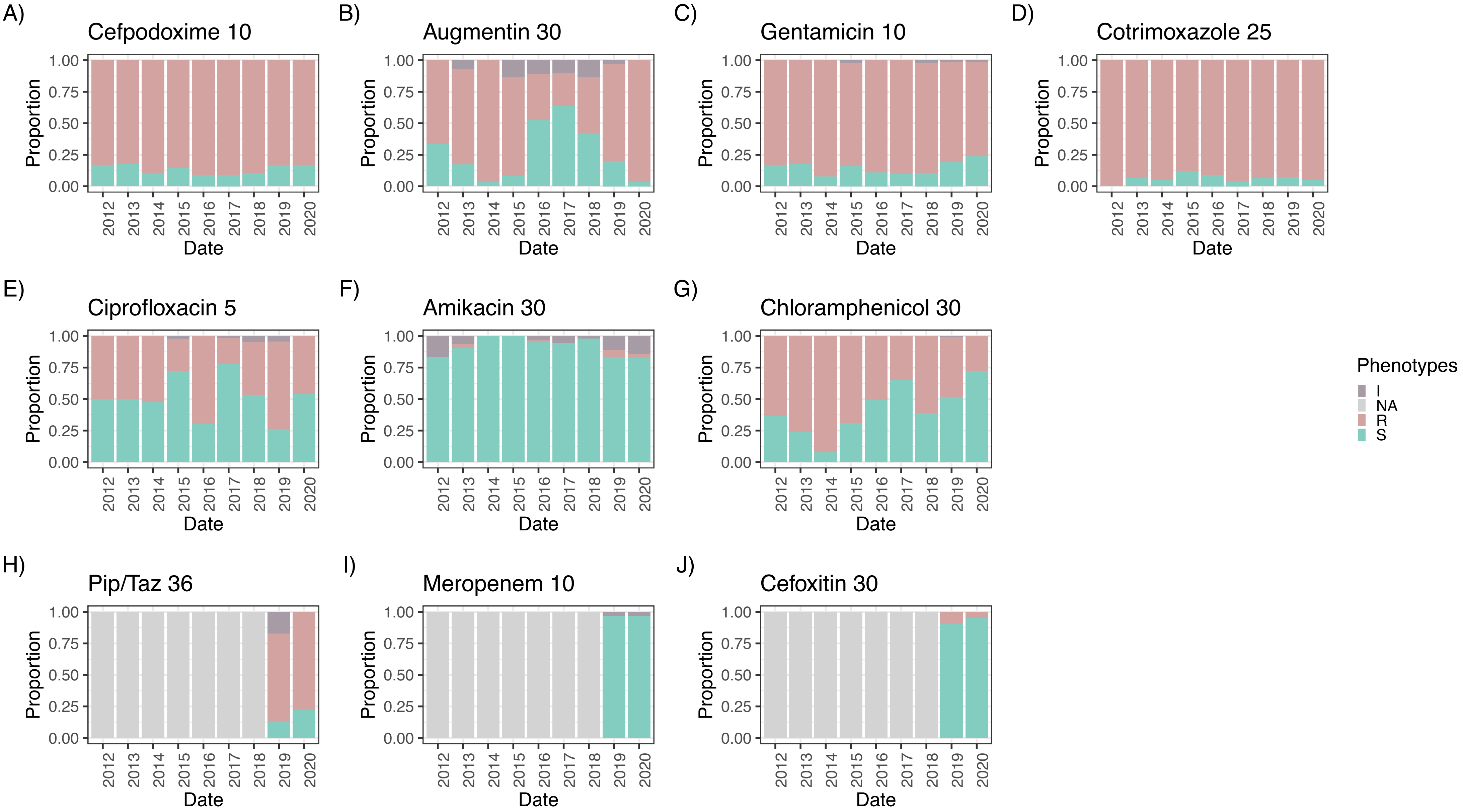

A)
Cefpodoxime 10
B)
Augmentin 30
C)
Gentamicin 10
D)
Cotrimoxazole 25
E)
Ciprofloxacin 5
F)
Amikacin 30
G)
Chloramphenicol 30
H)
Pip/Taz 36
I)
Meropenem 10
J)
Cefoxitin 30
Proportion
0.00
0.25
0.50
0.75
1.00
Date
2012
2013
2014
2015
2016
2017
2018
2019
2020
Phenotypes
I
NA
R
S

Fig S4. Phenotypic resistance profiles of all sequenced isolates. This highlights very high resistance against widely used first-line treatments like (**A**) cefpodoxime (ceftriaxone; 3GC), (**B**) augmentin (BLBLI combination), (**C**) gentamicin (AGly), (**D**) cotrimoxazole (TMT) but still a large proportion of sensitive isolates against less commonly used antimicrobials; (**E**) ciprofloxacin (Fq), (**F**) amikacin (AGly), and a pattern of increasing sensitivity against (**G**) chloramphenicol. The currently only available last-line treatments are (**H**) piperazillin-tazobactam (BLBLI combination), which show very high resistance levels; and (**I**) meropenem (carbapenem), the currently only viable alternative tested. (**J**) Cefoxitin also shows a lot of sensitive isolates; this antimicrobial is routinely used in diagnostic laboratories world-wide to test for *ampC* production as it was rapidly replaced with third-generation cephalosporins after its initial characterisation. The resistance profile is however of high interest as clinical trials have been started recently to assess it as a potential treatment alternative.

Figure S5A

K. pneumoniae subsp. pneumoniae

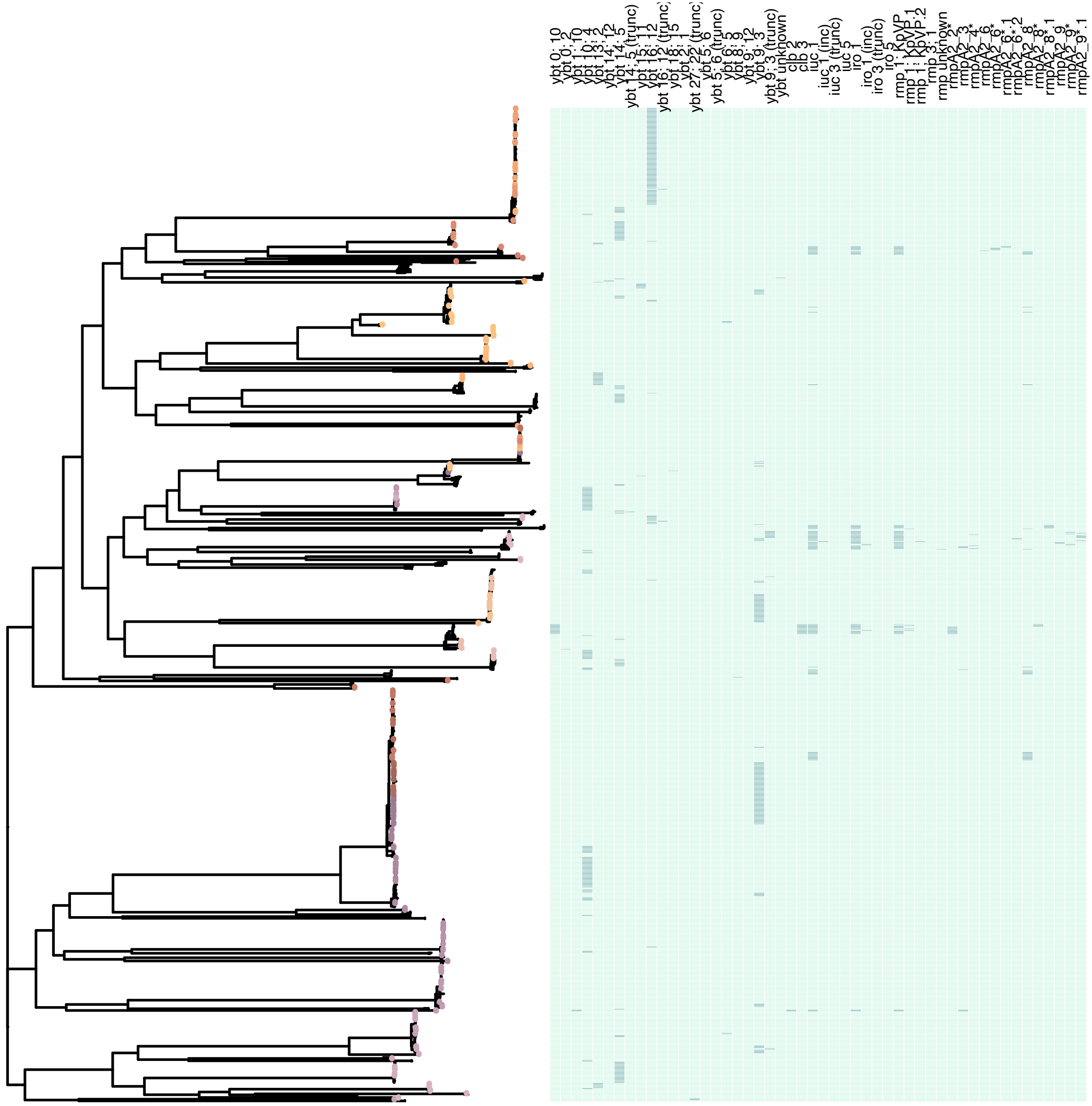

Figure S5B

K. pneumoniae subsp. pneumoniae

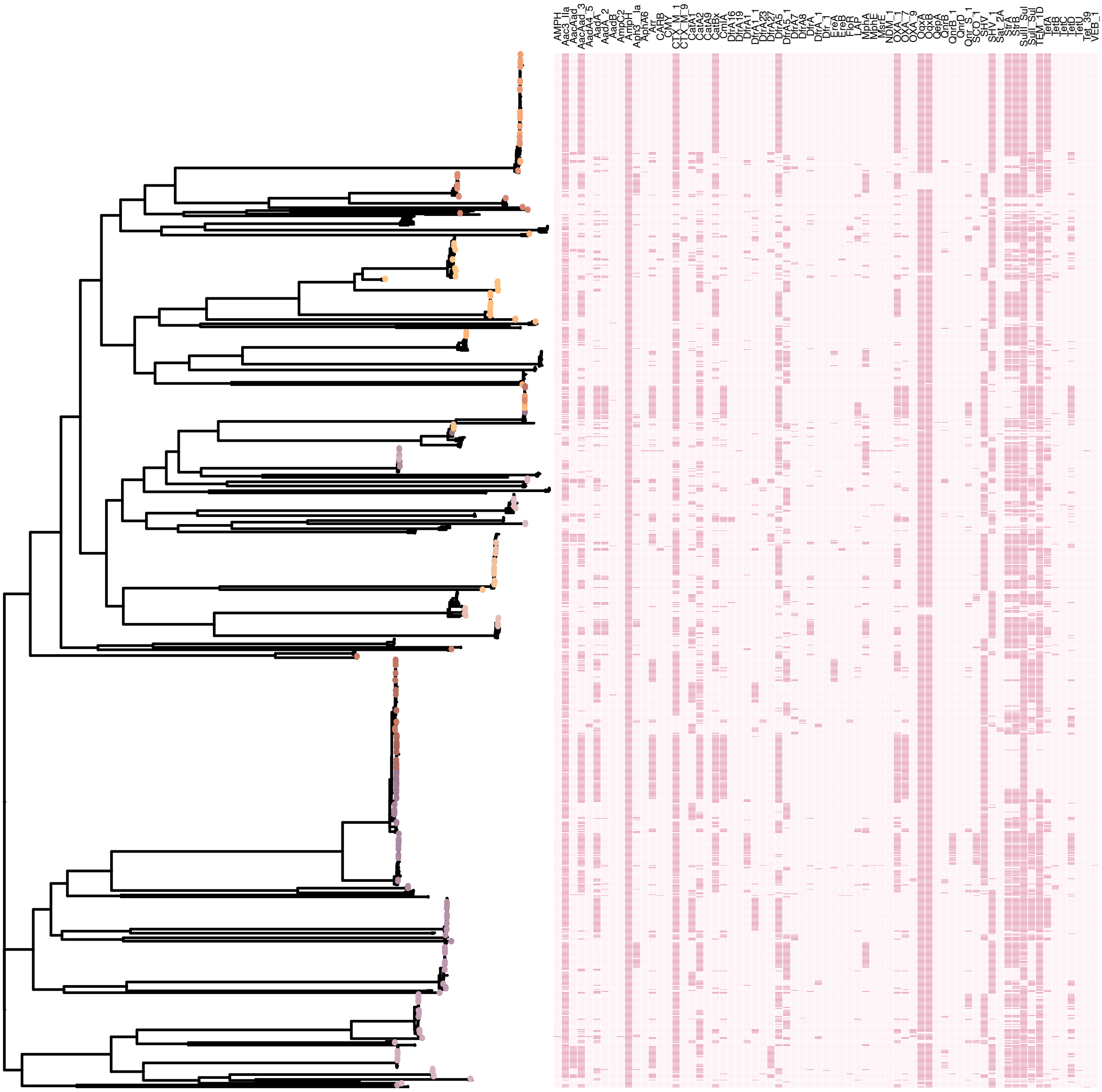

Figure S5C

K. pneumoniae subsp. pneumoniae

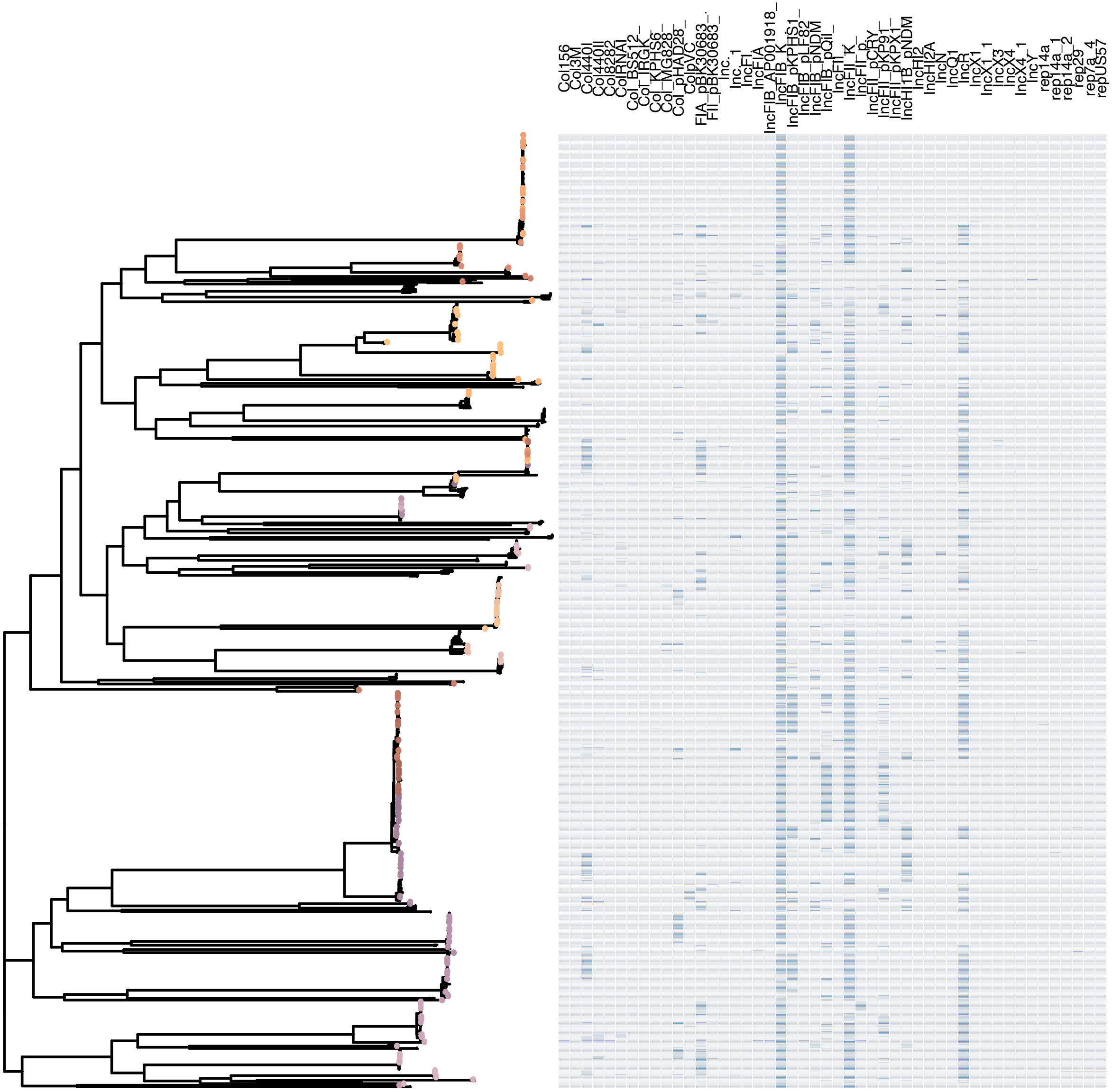

Fig S5. Predicted resistance and virulence genes and plasmid replicons for *K. pneumoniae*. The guidance tree is as shown in Fig. 3A, highlighting closely related isolates by tip labels. The heatmaps show the presence (dark shade) or absence (light shade) of (**A**) virulence genes, (**B**) resistance genes and (**C**) plasmid replicons.

Figure S6

A)

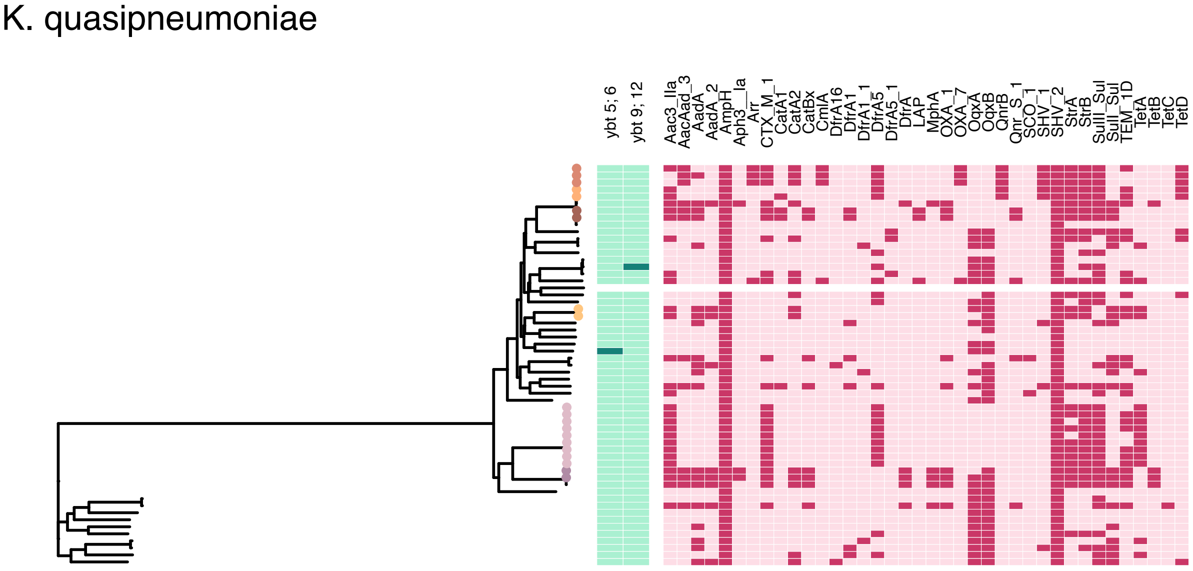
K. quasipneumoniae
ybt 5; 6
ybt 9; 12
Aac3_IIa
AacAad_3
AadA
AadA_2
AmpH
Aph3__Ia
Arr
CTX_M_1
CatA1
CatA2
CatBx
CmlA
DfrA16
DfrA1
DfrA1_1
DfrA5
DfrA5_1
DfrA
LAP
MphA
OXA_1
OXA_7
OqxA
OqxB
QnrB
Qnr_S_1
SCO_1
SHV_1
SHV_2
StrA
StrB
SulII_Sul
SulI_Sul
TEM_1D
TetA
TetB
TetC
TetD

B)

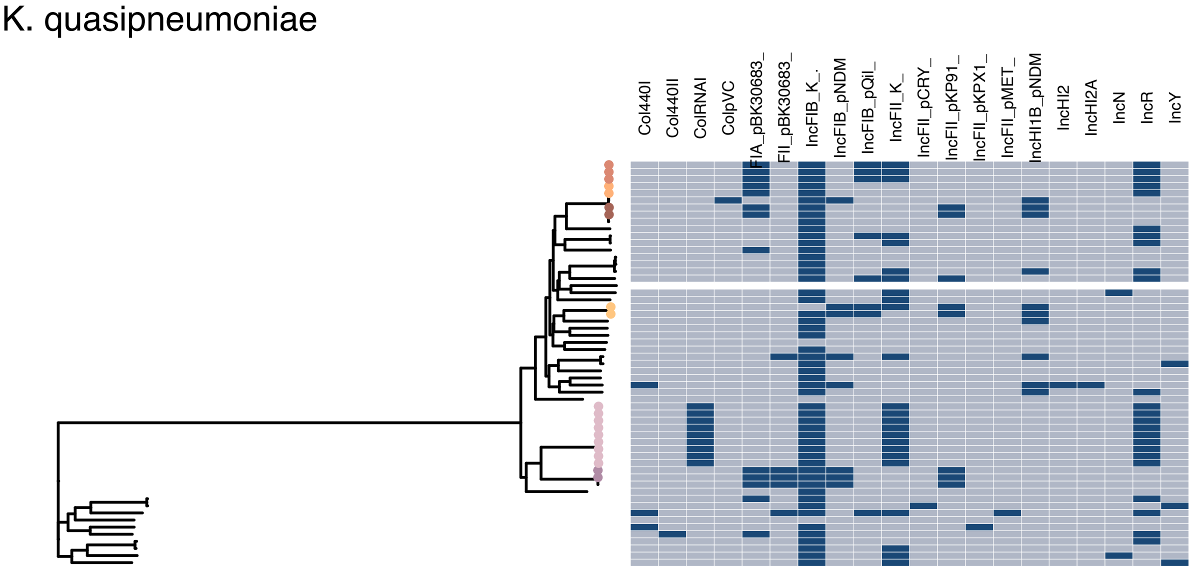
K. quasipneumoniae
Col440I
Col440II
ColRNAI
ColpVC
FIA_pBK30683_
FII_pBK30683_
IncFIB_K_
IncFIB_pNDM
IncFIB_pQil_
IncFII_K_
IncFII_pCRY_
IncFII_pKP91_
IncFII_pKPX1_
IncFII_pMET_
IncHI1B_pNDM
IncHI2
IncHI2A
IncN
IncR
IncY

Fig S6. Predicted resistance and virulence genes and plasmid replicons for *K. quasipneumoniae*. The guidance tree is as shown in Fig. 3B, highlighting closely related isolates by tip labels. The heatmaps show the presence (dark shade) or absence (light shade) of (**A**) virulence and resistance genes and (**B**) plasmid replicons.

Figure S7

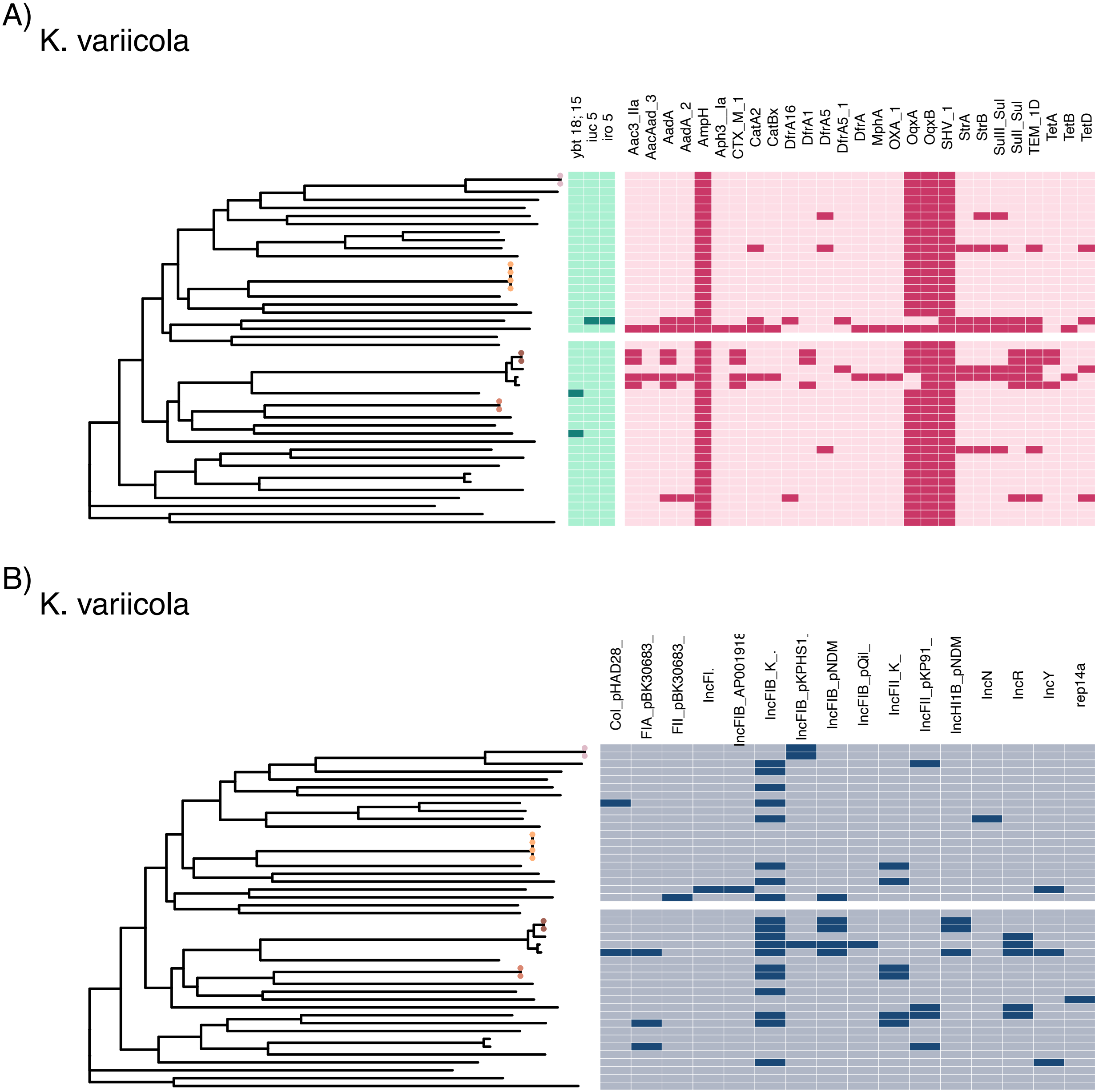
A)
K. variicola
ybt 18; 15
iuc 5
iro 5
Aac3_IIa
AacAad_3
AadA
AadA_2
AmpH
Aph3__Ia
CTX_M_1
CatA2
CatBx
DfrA16
DfrA1
DfrA5
DfrA5_1
DfrA
MphA
OXA_1
OqxA
OqxB
SHV_1
StrA
StrB
SulII_Sul
SulI_Sul
TEM_1D
TetA
TetB
TetD
B)
K. variicola
Col_pHAD28_
FIA_pBK30683_
FII_pBK30683_
IncFI.
IncFIB_AP001918
IncFIB_K_.
IncFIB_pKPHS1_
IncFIB_pNDM
IncFIB_pQil_
IncFII_K_
IncFII_pKP91_
IncHI1B_pNDM
IncN
IncR
IncY
rep14a

Fig S7. Predicted resistance and virulence genes and plasmid replicons for *K. variicola*. The guidance tree is as shown in Fig. 3C, highlighting closely related isolates by tip labels. The heatmaps show the presence (dark shade) or absence (light shade) of (**A**) virulence and resistance genes and (**B**) plasmid replicons.

Figure S8

A)

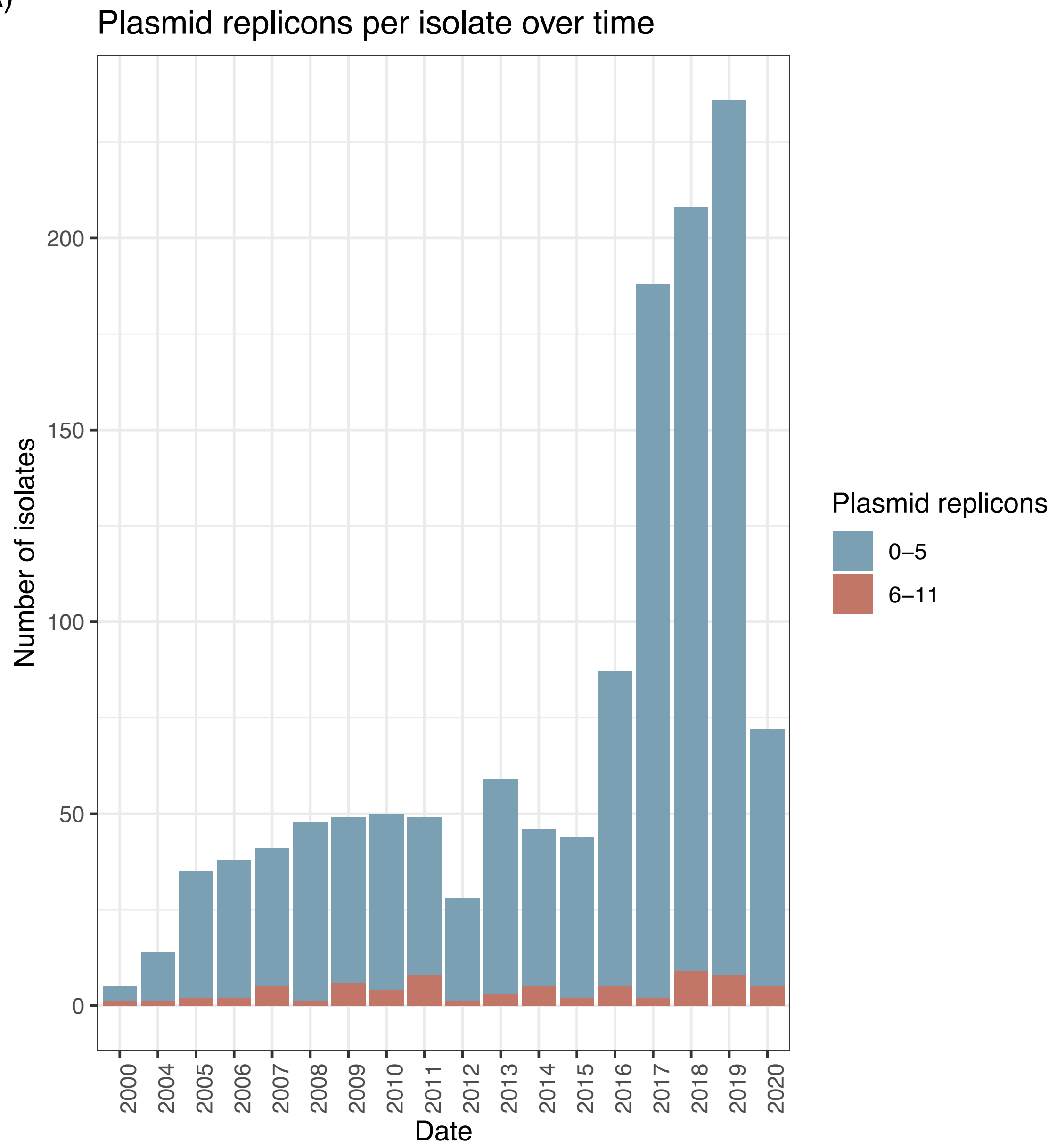
Plasmid replicons per isolate over time
Number of isolates
0
50
100
150
200
2000
2004
2005
2006
2007
2008
2009
2010
2011
2012
2013
2014
2015
2016
2017
2018
2019
2020
Date
Plasmid replicons
0–5
6–11

B)

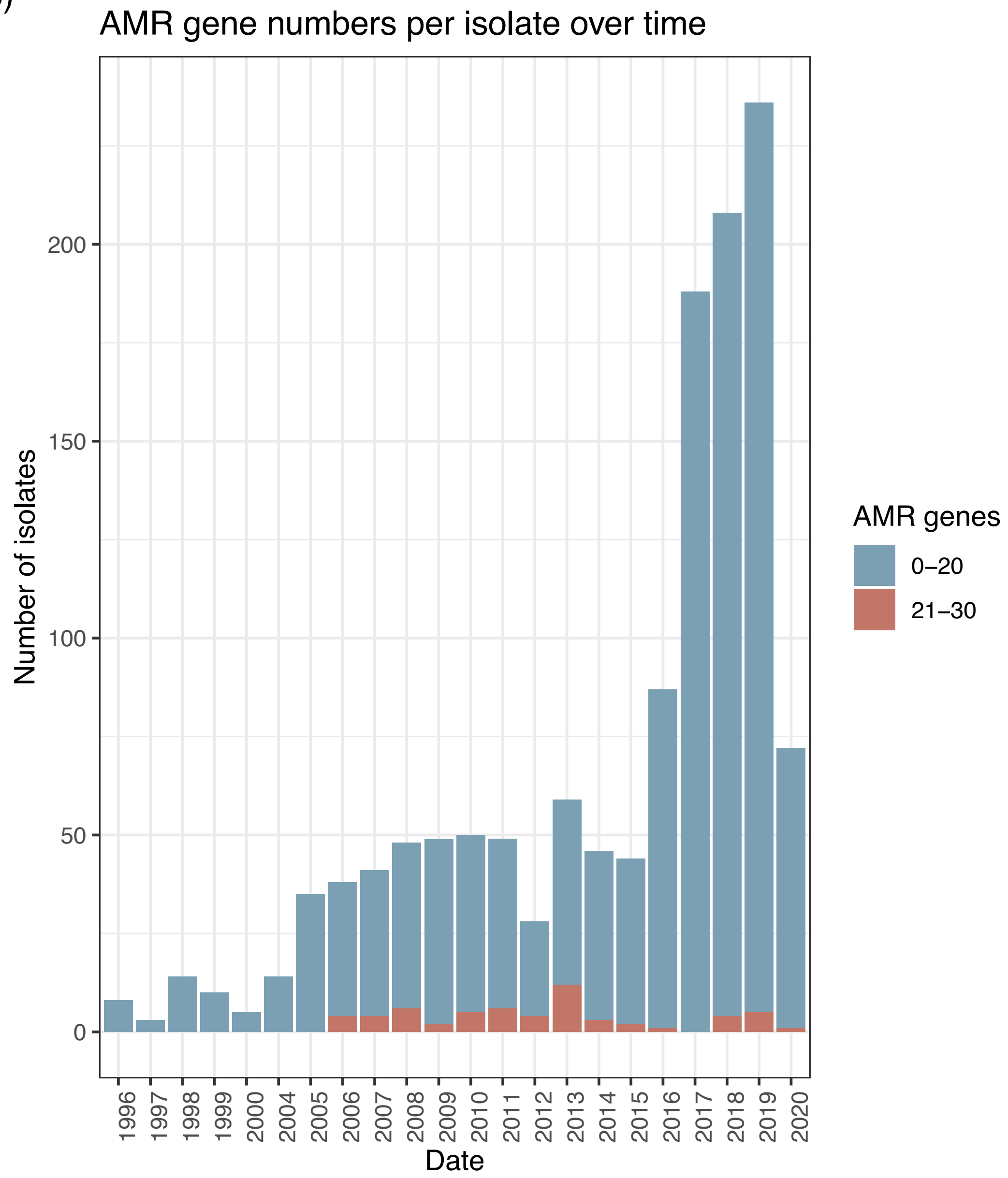
AMR gene numbers per isolate over time
Number of isolates
0
50
100
150
200
1996
1997
1998
1999
2000
2004
2005
2006
2007
2008
2009
2010
2011
2012
2013
2014
2015
2016
2017
2018
2019
2020
Date
AMR genes
0–20
21–30

Fig S8. Plasmid replicons and resistance gene numbers. (**A**) *Kpn* isolates over time showing the number of plasmid replicons encoded, and (**B**) the number of isolates for numbers of resistance genes present over time.

Figure S9

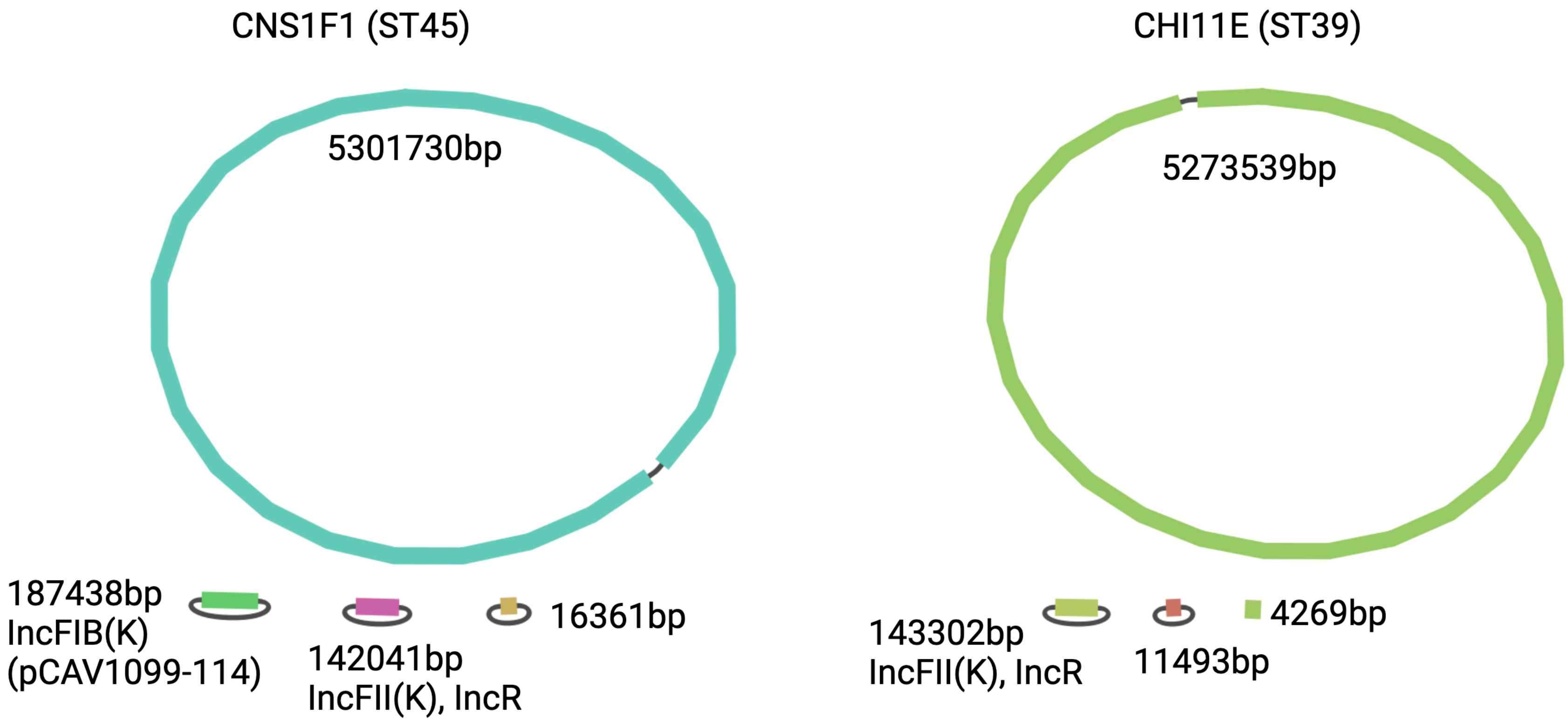
CNS1F1 (ST45)
5301730bp
187438bp
IncFIB(K)
(pCAV1099-114)
142041bp
IncFII(K), IncR
16361bp
CHI11E (ST39)
5273539bp
143302bp
IncFII(K), IncR
11493bp
4269bp

Fig S9. Visualisation of assembly graphs as generated by flye using bandage. The assembled contigs are illustrated and show the length in bp after polishing steps as well as the plasmid replicons as predicted by the online plasmidfinder platform.

Figure S10

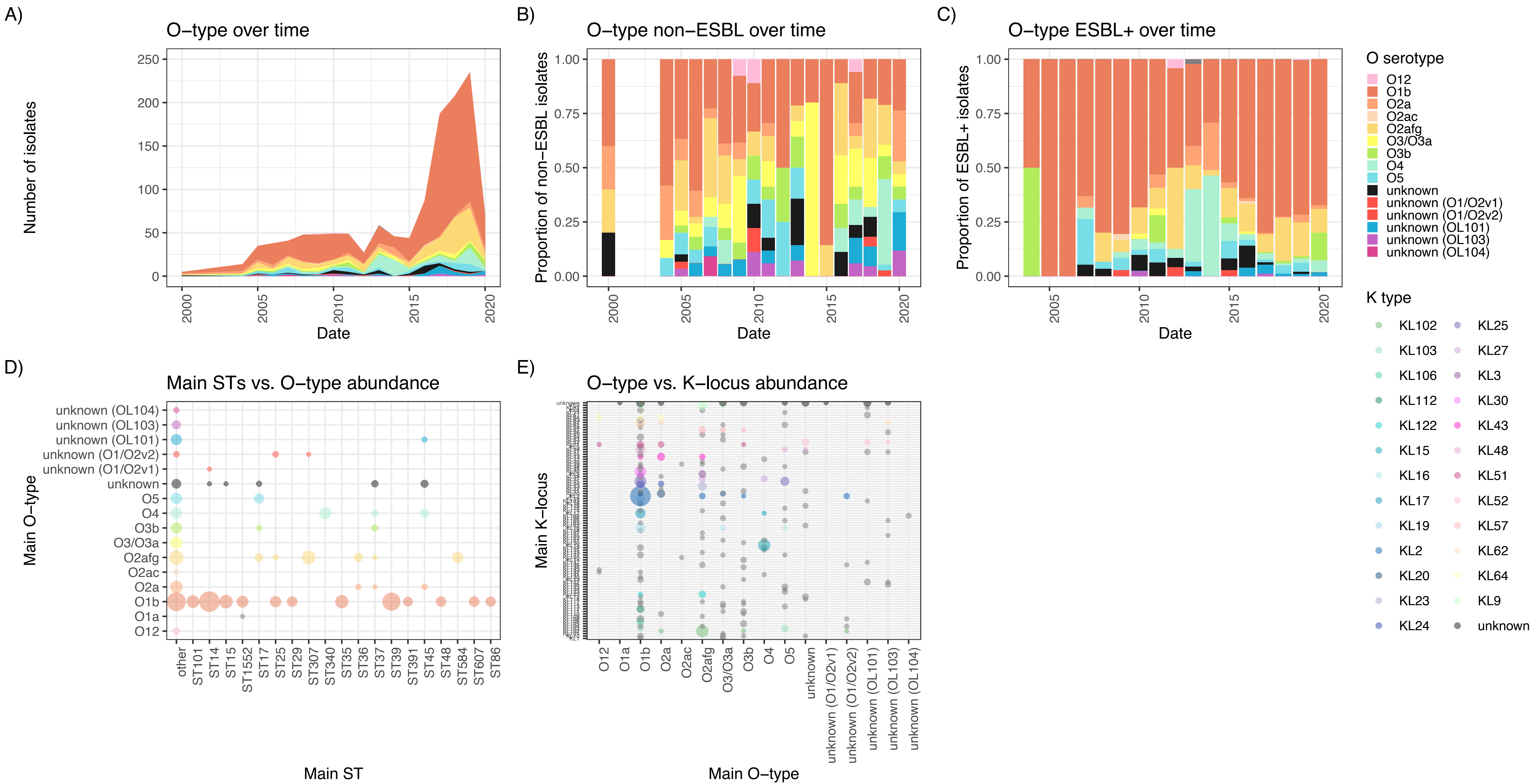

A)
O−type over time
Number of isolates
Date
B)
O−type non−ESBL over time
Proportion of non−ESBL isolates
Date
C)
O−type ESBL+ over time
Proportion of ESBL+ isolates
Date
O serotype
O12
O1b
O2a
O2ac
O2afg
O3/O3a
O3b
O4
O5
unknown
unknown (O1/O2v1)
unknown (O1/O2v2)
unknown (OL101)
unknown (OL103)
unknown (OL104)
K type
KL102
KL103
KL106
KL112
KL122
KL15
KL16
KL17
KL19
KL2
KL20
KL23
KL24
KL25
KL27
KL3
KL30
KL43
KL48
KL51
KL52
KL57
KL62
KL64
KL9
unknown
D)
Main STs vs. O−type abundance
Main O−type
Main ST
E)
O−type vs. K−locus abundance
Main K−locus
Main O−type

Fig S10. O-Ag serotypes over time and resistance profiles. The distribution of predicted O-Ag serotypes based on their genomic loci(24,27) over time for (**A**) total numbers of all isolates, (**B**) porportions of non-ESBL and (**C**) ESBL-encoding isolates; and their distribution across (**D**) main STs and (**E**) K-locus types.

Figure S11

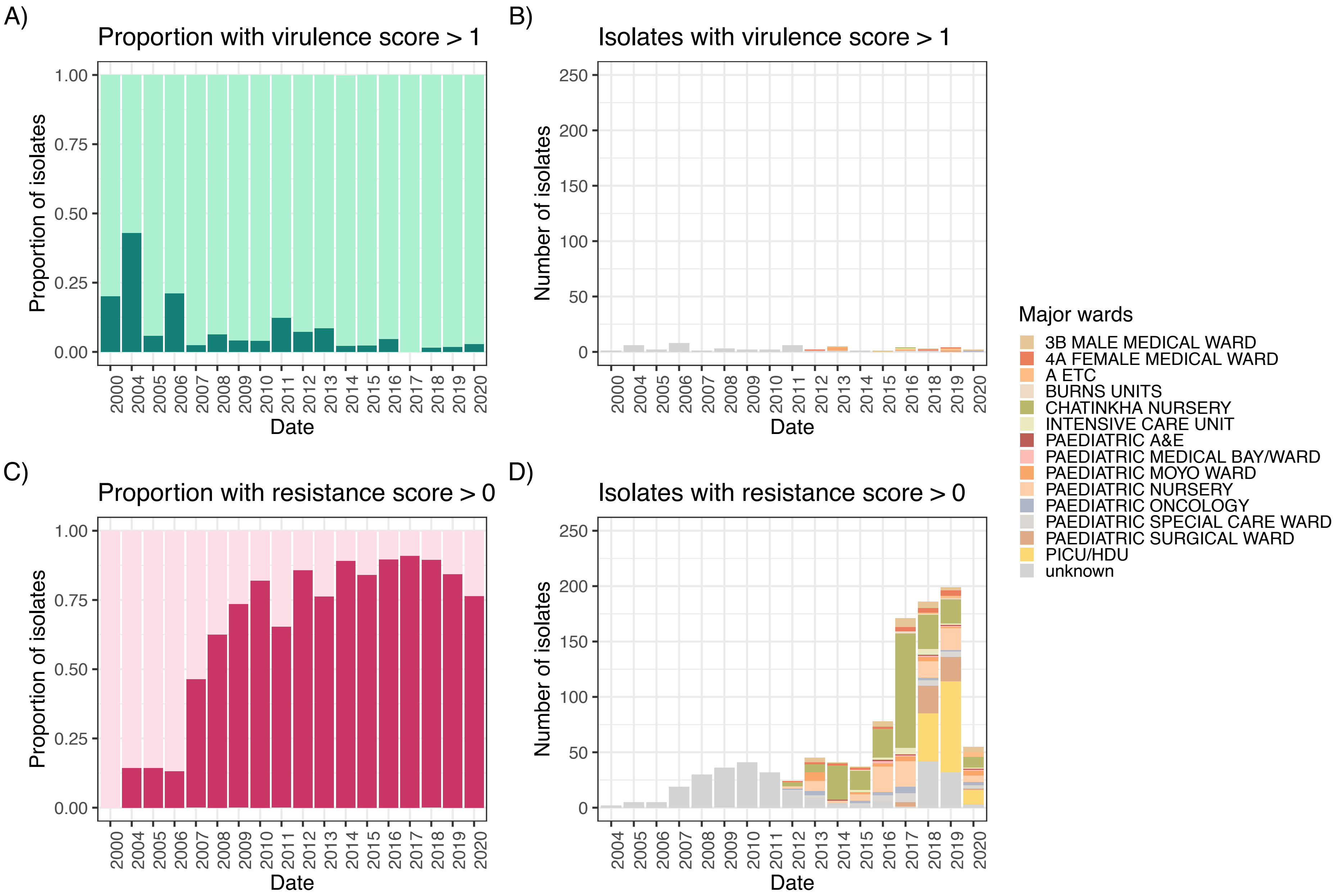
A)
Proportion with virulence score > 1
Proportion of isolates
Date
B)
Isolates with virulence score > 1
Number of isolates
Date
C)
Proportion with resistance score > 0
Proportion of isolates
Date
D)
Isolates with resistance score > 0
Number of isolates
Date
Major wards
3B MALE MEDICAL WARD
4A FEMALE MEDICAL WARD
A ETC
BURNS UNITS
CHATINKHA NURSERY
INTENSIVE CARE UNIT
PAEDIATRIC A&E
PAEDIATRIC MEDICAL BAY/WARD
PAEDIATRIC MOYO WARD
PAEDIATRIC NURSERY
PAEDIATRIC ONCOLOGY
PAEDIATRIC SPECIAL CARE WARD
PAEDIATRIC SURGICAL WARD
PICU/HDU
unknown

Fig S11. Proportions of virulent isolates and resistant isolates over time have markedly different pattern. (**A**) The number of isolates with a kleborate-predicted virulence score > 1 (1 usually indicates yersiniabactin which is chromosomally fixed in several STs) which remains very low and stable over time and found sporadically across various wards, whereas (**B**) isolates with resistance scores 1 or over (indicating ESBL or, in the case of score of 2, carbapenemase present) rapidly increase over time and biased towards several wards. This is reflected in the proportions; whilst isolates encoding several virulence factors remain at very low proportion over time (**C**), we can see the rapid increase of resistant isolates which remains at a very high level since the initial increase (**D**).

Figure S12

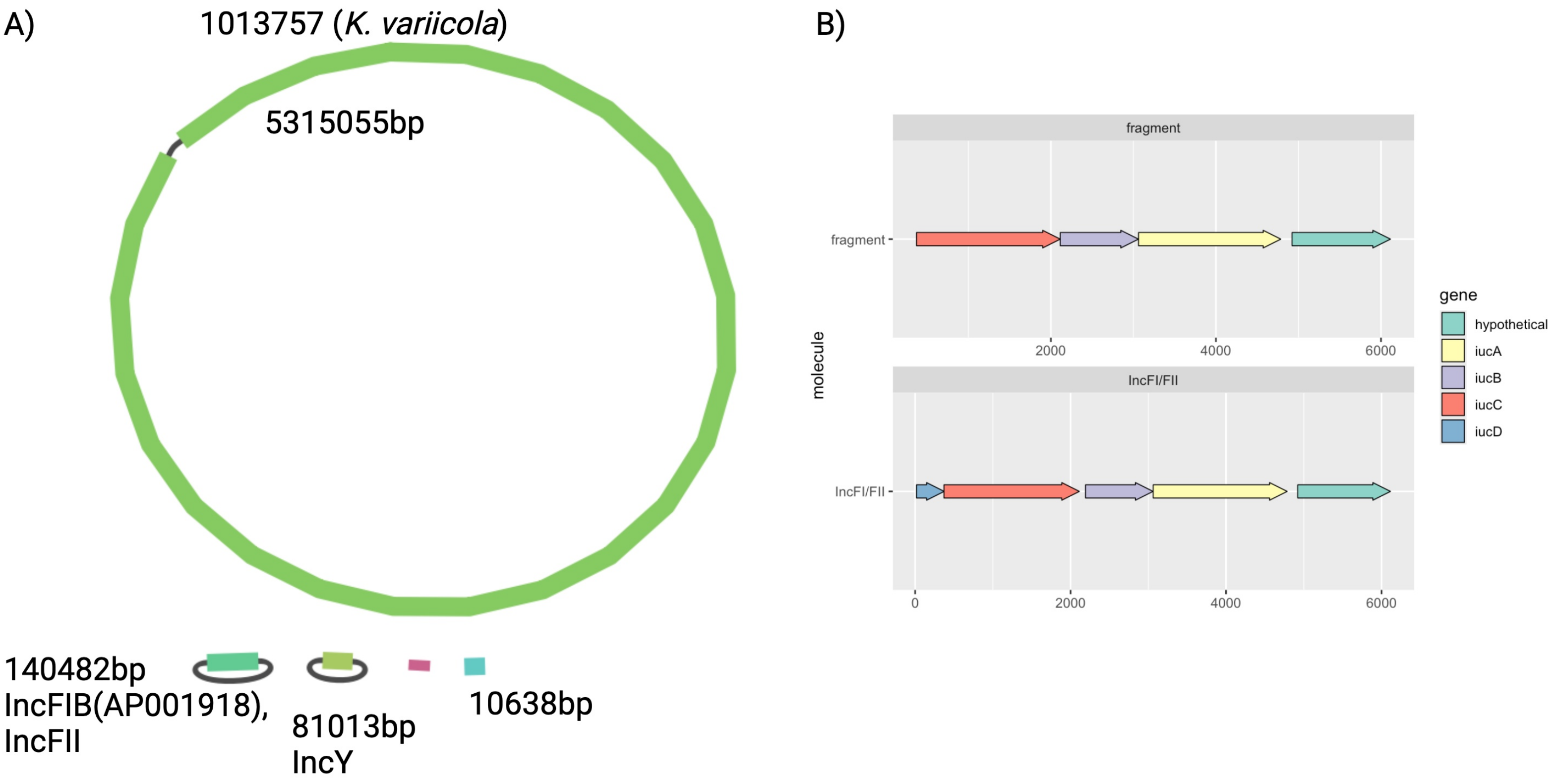
A)
1013757 (*K. variicola*)
5315055bp
140482bp
IncFIB(AP001918),
IncFII
81013bp
IncY
10638bp
B)
fragment
fragment
2000
4000
6000
molecule
IncFI/FII
IncFI/FII
0
2000
4000
6000
gene
hypothetical
iucA
iucB
iucC
iucD

Fig S12. Visualisation of assembly graphs as generated by flye using bandage. The assembled contigs are illustrated and show the length in bp after polishing steps as well as the plasmid replicons as predicted by the online plasmidfinder platform (**A**) and the predicted virulence-gene operon is shown in (**B**) as assembled. The virulence operon is at the contig end of the IncFI/FII plasmid and thus also misassembled into a small fragment, reflective of the challenges in assembling mobile elements.

Table S1: Accessions, metadata and kleborate predictions per strain.
Table S2: Antimicrobial gene predictions using ariba.
Table S3: Plasmid replicon predictions using ariba.
Table S4: Phenotypic resistance profiles.
Table S5: O- and K-antigen predictions using updated definitions in kaptive.
Table S6: Long-read sequencing assembly description and accessions.
